# Vitamin C and vitamin D_3_ alleviate metabolic-associated fatty liver disease by regulating the gut microbiota and bile acid metabolism *via* the gut-liver axis

**DOI:** 10.3389/fphar.2023.1163694

**Published:** 2023-04-05

**Authors:** Qingling Chen, Lili Zhao, Ling Mei, Xiaotong Zhao, Ping Han, Jie Liu, Chao Meng, Ruifang Li, Rui Zhong, Kai Wang, Jia Li

**Affiliations:** ^1^ Clinical School of the Second People’s Hospital, Tianjin Medical University, Tianjin, China; ^2^ Department of Gastroenterology and Hepatology, Tianjin Second People’s Hospital, Tianjin, China; ^3^ Department of Clinical Laboratory, Tianjin Second People’s Hospital, Tianjin, China; ^4^ School of Medicine, Nankai University, Tianjin, China; ^5^ Department of Neurology, The First Hospital of Jilin University, Changchun, Jilin, China; ^6^ Key Laboratory of Bioactive Materials for the Ministry of Education, College of Life Sciences, Nankai University, Tianjin, China

**Keywords:** metabolic-associated fatty liver disease, vitamin C, vitamin D3, gut microbiota, bile acid metabolism, gut-liver axis

## Abstract

**Background:** Previous studies have demonstrated that both vitamin C (VC) and vitamin D_3_ (VD_3)_ have therapeutic potential against metabolic disorders, including obesity, diabetes, and metabolic-associated fatty liver disease (MAFLD). However, it is unclear whether VC supplementation is associated with improving the intestinal flora and regulating the metabolism of bile acids *via* the gut-liver axis in MAFLD. There is still no direct comparison or combination study of these two vitamins on these effects.

**Methods:** In this study, we employed biochemical, histological, 16S rDNA-based microbiological, non-targeted liver metabolomic, and quantitative real-time polymerase chain reaction analyses to explore the intervening effect and mechanism of VC and VD_3_ on MAFLD by using a high-fat diet (HFD)-induced obese mouse model.

**Results:** Treatment of mice with VC and VD_3_ efficiently reversed the characteristics of MAFLD, such as obesity, dyslipidemia, insulin resistance, hepatic steatosis, and inflammation. VC and VD_3_ showed similar beneficial effects as mentioned above in HFD-induced obese mice. Interestingly, VC and VD_3_ reshaped the gut microbiota composition; improved gut barrier integrity; ameliorated oxidative stress and inflammation in the gut-liver axis; inhibited bile acid salt reflux-related ASBT; activated bile acid synthesis-related CYP7A1, bile acid receptor FXR, and bile acid transportation-related BSEP in the gut-liver axis; and improved bile secretion, thus decreasing the expression of FAS in the liver and efficiently ameliorating MAFLD in mice.

**Conclusion:** Together, the results indicate that the anti-MAFLD activities of VC and VD_3_ are linked to improved gut-liver interactions *via* regulation of the gut microbiota and bile acid metabolism, and they may therefore prove useful in treating MAFLD clinically.

## Introduction

Metabolic-associated fatty liver disease (MAFLD), formerly named non-alcoholic fatty liver disease (NAFLD), the prevalence of which has increased alarmingly, places a significant burden on the health and economy of all societies (Eslam et al., 2020a; Sarin et al., 2020). The use of the new term MAFLD is strongly supported by many countries and regions (Zhang et al., 2022; Chung et al., 2023; Lutsiv et al., 2023). According to a review and meta-analysis conducted recently, the prevalence of MAFLD in Asia is 29.62% (Li et al., 2019). In addition to lipid accumulation, MAFLD is associated with oxidative stress, lobular inflammation, apoptosis, and fibrosis (Friedman et al., 2018). The growth of this disease has been fueled by excess high calorie energy intake, sedentary behavior, and low levels of physical activity. Thus, the primary therapy for MAFLD remains lifestyle changes, including dietary control, weight loss, and exercise intervention (Eslam et al., 2020b). Although there are many drugs in the pipeline that are considered good candidates for curing MAFLD/MASH (Negi et al., 2022), there are no approved pharmacotherapies for MAFLD as of yet, highlighting the urgent need to develop new preventive and therapeutic strategies to manage MAFLD.

Within the gut, approximately 1 × 1,014 microbial cells make up the intestinal microbiota and function as a metabolic and endocrine organ (Heintz-Buschart and Wilmes, 2018). Under normal conditions, commensal bacteria promote the health of the host in multiple ways, from assisting with digestion to producing vitamins and enhancing immune system homeostasis (Round and Mazmanian, 2009). In recent years, the intestinal microbiome has gained a great deal of attention, as it plays a significant role in regulating host substrate metabolism as well as energy metabolism in obesity and MAFLD (Sommer and Backhed, 2013; Rao et al., 2021; Sang et al., 2021). The function of bile acid is to facilitate digestion, the absorption of lipids and vitamins, and signal transduction. Many bacterial species are involved in the metabolism of bile acids. It may be possible that probiotics that modulate bile acid levels and nuclear receptor activity may help restore the gut microbiota and improve MAFLD (Chen et al., 2019).

As a water-soluble vitamin, vitamin C (VC) has multiple pharmacological effects and antioxidant properties, including the ability to scavenge reactive oxygen species and to reduce oxidative stress *in vivo* and *in vitro* (Padayatty et al., 2003; Khoshnam-Rad and Khalili, 2019). Our previous study showed that medium-dose VC (30 mg/kg per day) administration could be beneficial for both prophylaxis and the treatment of MAFLD through adiponectin/adipoRII signaling (Zeng et al., 2020). Lee et al. reported that mega-dose VC treatment suppresses high-fructose diet-mediated MAFLD progression by decreasing diet ingestion and increasing water intake (Lee et al., 2022). Vitamin D (VD) is a fat-soluble vitamin that is closely associated with health (Jukic et al., 2018). There is accumulating evidence that VD affects the metabolism of glucose and lipids in MAFLD by reducing inflammation, reducing antioxidant damage, and enhancing insulin sensitivity (Erbas et al., 2014; Barchetta et al., 2020; Dong et al., 2020). Moreover, VD has been shown to restore high-fat diet (HFD)-induced gut microbiota dysbiosis, maintain the mucosal barrier, and modulate immune-metabolic pathways within the gut–liver axis (Su et al., 2016; Jahn et al., 2019; Zhang et al., 2021). In addition, one recent study demonstrated that VC combined with VD_3_ can regulate the expression of claudin-2 and promote mucosal barrier repair in ulcerative colitis (Qiu et al., 2021). However, it is unclear whether supplementation with VC improves intestinal flora and regulates bile acid metabolism *via* the gut-liver axis in MAFLD. A direct comparison and examination of the combined use of these two vitamins on these effects are still lacking.

Contextually, the use of natural products to cure/alleviate this very common liver disease is appealing to physicians, based on the abundance of literature data (Tarantino et al., 2021). In the present study, we employed biochemical, histological, 16S rDNA-based microbiological, non-targeted liver metabolomic, quantitative real-time polymerase chain reaction (qRT‒PCR), and immunohistochemical analyses to assess the influence of VC and VD_3_ on MAFLD in HFD-induced obese mice. The aim of the current study was to determine how the gut microbiota and liver metabolome are modulated by VC and VD_3_.

## Materials and methods

### Materials

Vitamin C (Tianjin Kingyork, H12020392), Vitamin D3 (Beijing Solarbio Science & Technology, V8070), Mouse TNF-alpha ELISA Kit (RayBiotech, P06804), Mouse IL-6 ELISA Kit (RayBiotech, P08505), Mouse IL-1β ELISA Kit (Shanghai Tongwei Biotechnology, TW56961), Mouse insulin ELISA Kit (Shanghai Tongwei Biotechnology, TW085566), Mouse endotoxin (ET) ELISA Kit (Jiangsu Meimian, MM-0369M1), Total Cholestenone Content Assay Kit (Beijing Solarbio Science & Technology, BC 1985), Triglyceride Content Assay Kit (Beijing Solarbio Science & Technology, BC0625), Total SOD activity detection kit (Shanghai Beyotime Biotechnology, S0101S), Lipid oxidation detection kit (Shanghai Beyotime Biotechnology, S0131S), BCA protein concentration assay kit (Shanghai Beyotime Biotechnology, P0010), Oil red O (Sigma, O0625), Anti-FXR1 antibody (Abcam, ab155124), BSEP Monoclonal antibody (Proteintech Group, 67512-1-Ig), Anti-Fas antibody (Abcam, ab271016), Anti-ZO1 tight junction protein antibody (Abcam, ab221547), Anti-Occludin antibody (Abcam, ab222691), Anti-SLC10A2/ASBT antibody (Abcam, ab203205), TRIzol Reagent (Thermo Fisher, 15596026), cDNA synthesis kit (Beijing Tiangen Biotech, KR118), and SuperReal PreMix Plus (SYBR Green) (Beijing Tiangen Biotech, FP205) were used.

### VC and VD_3_ preparation

As a pretreatment for VC solution, we added 32 mL of normal saline (NS) to dissolve 100 mg (1 mL) of VC to prepare a final concentration of 3.0 mg/mL VC solution. VC solution should be prepared when used, and the liquid emulsion should not be kept for too long after it is prepared. To pretreat VD_3_, we dissolved 10 mg of VD_3_ powder in 1 mL of absolute ethanol and then diluted it with 499 mL of NS to produce a 20 μg/mL solution, which was stored at 4°C in the dark (prepared once every 3 days), and then the final concentration of 0.2 μg/mL VD_3_ solution was prepared by diluting with NS.

### Animal experiments

The animal study was reviewed and approved by the Ethics Committee of Nankai University and was carried out in accordance with the Guide for the Care and Use of Laboratory Animals of the National Institutes of Health (No. 2021-SYDWLL-000338). Specific pathogen-free (SPF) male C57BL/6 mice (7-8 weeks, 20 ± 2 g) were purchased from the National Institutes for Food and Drug Control (Beijing, China) and were housed in an animal room of the Institute of Radiation Medicine, Chinese Academy of Medical Sciences (Tianjin, China) under a controlled temperature of 22°C ± 1°C and a 12-h light/dark cycle with free access to food and water.

One week after acclimatization, the mice were randomly divided into five groups (*n* = 12 in each group): the normal diet (ND) group, HFD group, VC group, VD group, and VC + VD group. The mice received diets from Beijing Keao Xieli Feed Co., Ltd. (Beijing, China). The ND group was fed a ND, while the other groups were fed a HFD (D12492) for 16 weeks. In addition, the VC group was prophylactically supplied with VC (30 mg/kg per day), the VD group was prophylactically supplied with VD_3_ (2 μg/kg per day), and the VC + VD group was prophylactically supplied with VC (30 mg/kg per day) and VD_3_ (2 μg/kg per day). The doses of both vitamins were chosen based on the doses described in the literature for use in mice (Yin et al., 2012; Manna et al., 2017; Zeng et al., 2020; Zhang et al., 2021). The ND group and HFD group were administered the same volume of NS. Every week, the body weights and food intake of the mice were monitored.

At the end of the experiment, we collected fresh fecal samples in sterile freezing tubes, froze them in liquid nitrogen, and then stored them at −80°C until analysis. All mice were anesthetized with isoflurane after overnight fasting, and blood and tissue samples were collected. The serum was separated by centrifugation at 4,000 rpm for 10 min at 4°C and then stored at −80°C. Tissues, including the liver and ileum, were carefully excised and weighed. One portion of the tissues was fixed in 10% neutral buffered formalin fixative, and the rest were frozen immediately in liquid nitrogen and then stored at −80°C for further analysis. Additionally, we harvested and weighed the white adipose tissues of the epididymis and perirenal region. Weight gain = (week 16 body weight—week 0 body weight)/week 0 body weight × 100%, liver index = liver weight/body weight × 100%, perirenal fat index = perirenal fat weight/body weight × 100%, and epididymal fat index = epididymal fat weight/body weight × 100%.

### Biochemical analyses

Serum alanine aminotransferase (ALT), aspartate aminotransferase (AST), total cholesterol (TC), triglycerides (TG), high-density lipoprotein cholesterol (HDL-C), low-density lipoprotein cholesterol (LDL-C), and total bile acid (TBA) levels were measured using an automatic biochemical analyzer (Beckman Coulter AU5800, California, United States). The fasting blood glucose (FBG) levels were estimated using a OneTouch Select Simple^®^ glucometer and test strips (LifeScan, United Kingdom). Mouse serum insulin, endotoxin, and inflammatory cytokines (TNF-α, IL-1β, and IL-6) were determined by enzyme-linked immunosorbent assay (ELISA) according to the manufacturer’s instructions. Homeostasis model assessment of insulin resistance (HOMA-IR) = [fasting insulin (mIU/L)] × [FBG (mmol/L)]/22.5, and insulin sensitive index (ISI) = 1/[fasting insulin (mIU/L) ×FBG (mmol/L)].

With a high-speed low-temperature tissue grinding machine (Servicebio, Wuhan, China), 0.1 g of liver or ileum tissue was homogenized with 0.9 mL of phosphate buffered saline. The homogenate was centrifuged at 12,000 × g at 4°C for 10 min, and the supernatant was stored in sterile Eppendorf tubes for further analysis. Liver parameters, including TC, TG, endotoxin, superoxide dismutase (SOD), and malondialdehyde (MDA) levels, as well as ileum parameters, including endotoxin, SOD, and MDA levels, were determined using corresponding analytical reagent kits. All tests were performed according to the manufacturer’s protocols. The absorbance was read on a Biotek Synergy HT microplate reader. Each test was performed at least three times.

### Histological and immunohistochemical analyses

Formalin-fixed paraffin sections of the liver and ileum were cut at 5-μm thickness with a rotary microtome (Leica, Germany) before staining with hematoxylin-eosin (H&E), Masson’s trichrome, and immunohistochemistry. The liver tissues that were frozen in optimal cutting temperature compound (O.C.T.) were sectioned at 8-μm thickness and subjected to oil red O (ORO) to evaluate lipids. Representative images were captured by an upright optical microscope (Leica DM3000, Wetzlar, Germany) and assessed in a blinded manner as we previously reported (Zeng et al., 2020).

Liver expression of FXR, BSEP, and FAS and ileum expression of ZO-1, Occludin, and ASBT were detected using immunohistochemistry. Paraffin-embedded tissue samples were incubated with the corresponding primary antibody overnight at 4°C and then incubated with horseradish peroxidase-conjugated secondary antibody (Bioss, Beijing, China) for 1 h at room temperature according to the manufacturer’s recommendations. Brown staining was considered to be antibody-positive. The positive area was semiquantitatively analyzed by measuring the mean optical density of the brown areas with ImageJ 1.43 software.

### 16S rDNA amplicon sequencing

We extracted bacterial total genomic DNA from fecal samples using CTAB/SDS. The 16S rRNA genes in the 16S V4 region were amplified using specific primers (16S V4: 515F- 806R) and barcodes. We constructed sequencing libraries using the NEBNext^®^ Ultra™ IIDNA Library Prep Kit (Cat No. E7645) as directed by the manufacturer. Qubit@ 2.0 Fluorometer (Thermo Scientific) and Agilent Bioanalyzer 2,100 systems were used to evaluate the library quality. Finally, we sequenced the library using an Illumina NovaSeq platform and generated 250-bp paired-end reads. FLASH (Version 1.2.11, http://ccb.jhu.edu/software/FLASH/), fastp (Version 0.20.0), and Vsearch (Version 2.15.0) were used to obtain the Effective Tags from raw sequences by merging, quality filtering, and removing duplicates (Haas et al., 2011; Magoc and Salzberg, 2011). The effective tags obtained previously were denoised using DADA2 or the deblur module in quantitative insights into microbial ecology 2 (QIIME2) software (version QIIME2-202006) to obtain initial amplicon sequence variants (ASVs) (default: DADA2), and then ASVs with fewer than 5 reads were excluded (Li et al., 2020a). Reads were then performed using QIIME2 analysis.

### Hepatic untargeted metabolomics analysis

We ground tissues (0.1 g) individually with liquid nitrogen and then resuspended them with prechilled 80% methanol by vortexing. After incubation on ice for 5 min, the samples were centrifuged for 20 min at 15,000 × g and 4°C. A small amount of supernatant was used to dilute the final concentration of methanol to 53% using LC‒MS grade water. Following sample transfer to a new Eppendorf tube, the samples were centrifuged for 20 min at 15,000 × g and 4°C. Finally, the supernatant was injected into the LC‒MS/MS system for analysis (Want et al., 2013).

A Vanquish UHPLC system (Thermo Fisher, Germany) coupled with an Orbitrap Q ExactiveTM HF-X mass spectrometer (Thermo Fisher, Germany) was used by Novogene Co., Ltd. (Beijing, China) for UHPLC‒MS/MS analyses. On a Hypesil Gold column (100 × 2.1 mm, 1.9 μm), samples were injected using a 17-min linear gradient at a flow rate of 0.2 mL/min. A Q ExactiveTM HF-X mass spectrometer was operated in both positive and negative polarity modes with a spray voltage of 3.5 kV, capillary temperature of 320°C, sheath gas flow rate of 35 psi, aux gas flow rate of 10 L/min, S-lens RF level of 60, and Aux gas heater temperature of 350°C.

The raw data files generated by UHPLC‒MS/MS were processed using Compound Discoverer 3.1 (CD3.1, Thermo Fisher) to perform peak alignment, peak picking, and quantitation for each metabolite. For accurate qualitative and relative quantitative results, the peaks were matched with the mzCloud (https://www.mzcloud.org/), mzVault and MassList databases. These metabolites were annotated using the KEGG database (https://www.genome.jp/kegg/pathway.html), HMDB database (https://hmdb.ca/metabolites) and LIPIDMaps database (http://www.lipidmaps.org/). Based on the KEGG database, the functions of the identified differential metabolites and metabolic pathways were evaluated. An analysis of the data was conducted using the statistical software R (R version R-3.4.3), Python (Python 3.5.0 version), CentOS (CentOS release 6.6), and metaX (Wen et al., 2017).

### Total RNA extraction and qRT-PCR analysis

TRIzol reagent was used for total RNA isolation from tissues according to the manufacturer’s instructions. Using a cDNA synthesis kit, total RNA was reverse transcribed to cDNA. qRT‒PCR was carried out using SuperReal PreMix Plus (SYBR Green) on a 7,500 Real Time PCR System (ABI, United States). GAPDH was used as the reference gene. The relative fold change in mRNA expression was calculated using the 2^−ΔΔCT^ method. Table 1 shows the primer sequences synthesized by Sangon Biotech (Shanghai, China) for mouse primers.

**TABLE 1:** Primer sequences for quantification of the following target genes.

Target gene		Primer sequences (5′-3′)
Bacterial 16S		
V4 region	F	GTGCCAGCMGCCGCGGTAA (515F)
	R	GGACTACHVGGGTWTCTAAT (806R)
Mouse gene		
TLR4	F	ATG​GCA​TGG​CTT​ACA​CCA​CC
	R	GAG​GCC​AAT​TTT​GTC​TCC​ACA
Myd88	F	TCA​TGT​TCT​CCA​TAC​CCT​TGG​T
	R	AAA​CTG​CGA​GTG​GGG​TCA​G
CHOP	F	AAG​CCT​GGT​ATG​AGG​ATC​TGC
	R	TTC​CTG​GGG​ATG​AGA​TAT​AGG​TG
TNF-α	F	GAA​CCT​TTC​TGG​CCC​GTG​T
	R	AGA​AAT​CGC​AAT​TCA​TGT​CGC​A
IL-1β	F	GGTGCTGATGTACCAGTT
	R	TGGGCCTCAAAGGAAAGA
IL-6	F	TCT​ATA​CCA​CTT​CAC​AAG​TCG​GA
	R	GAA​TTG​CCA​TTG​CAC​AAC​TCT​TT
CYP7A1	F	GCT​GTG​GTA​GTG​AGC​TGT​TG
	R	GTT​GTC​CAA​AGG​AGG​TTC​ACC
FXR	F	GGC​AGA​ATC​TGG​ATT​TGG​AAT​CG
	R	GCC​CAG​GTT​GGA​ATA​GTA​AGA​CG
BSEP	F	TCT​GAC​TCA​GTG​ATT​CTT​CGC​A
	R	CCC​ATA​AAC​ATC​AGC​CAG​TTG​T
FAS	F	GCG​GGT​TCG​TGA​AAC​TGA​TAA
	R	GCA​AAA​TGG​GCC​TCC​TTG​ATA

### Statistical analysis

In all studies, one colleague was blinded to the experimental protocol. All data are shown as the mean ± SD. One-way ANOVA followed by Tukey’s *post hoc* test was used to identify differences between multiple groups. A *p*-value < 0.05 was considered to be statistically significant. All statistical analyses were carried out using GraphPad Prism 9.0.0. These data are representative of more than five biological replicates and three analytical replicates per biological replicate.

## Results

### VC and VD_3_ attenuated MAFLD in obese mice

**FIGURE 1:**
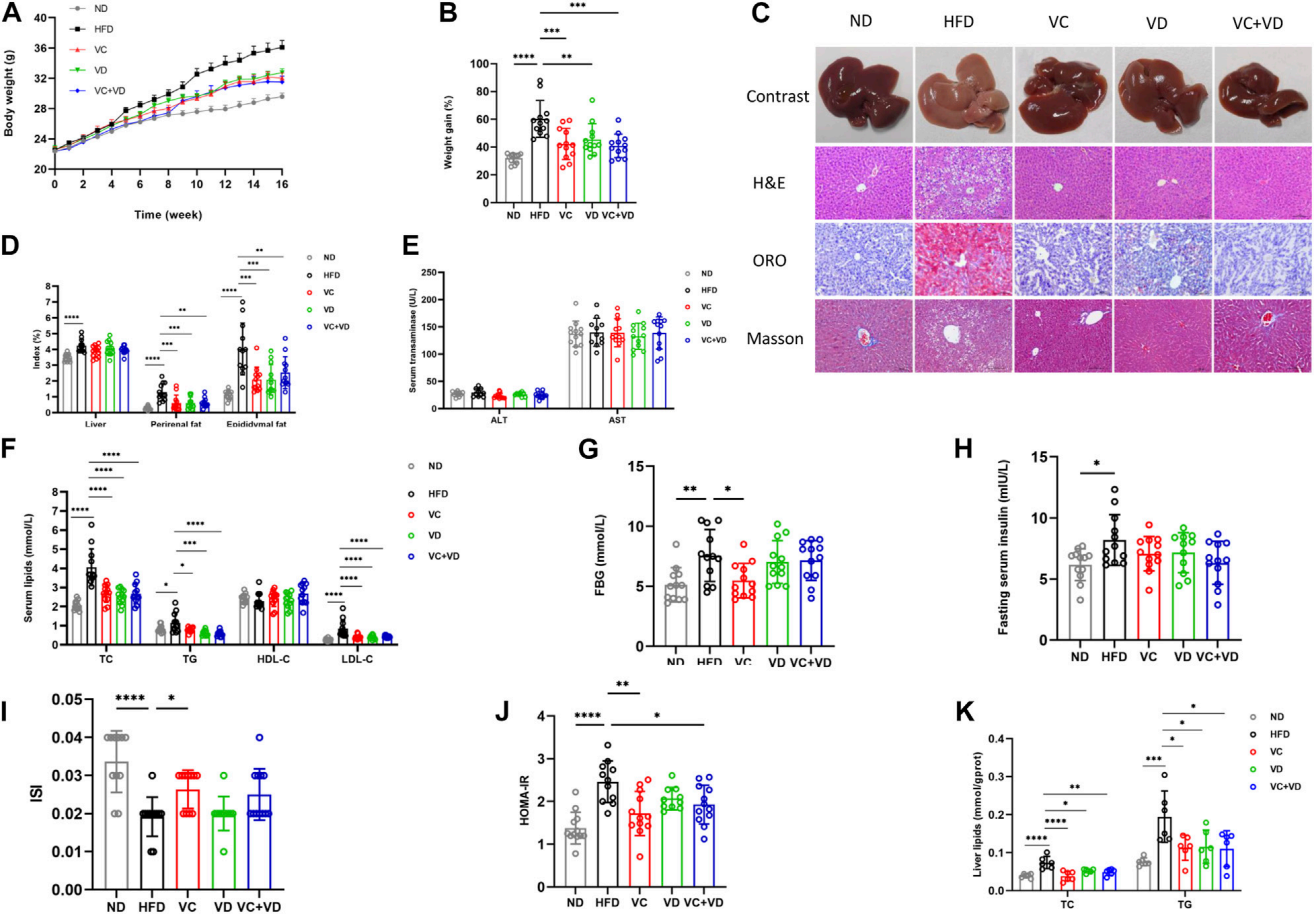
VC and VD_3_ attenuated MAFLD in obese mice. **(A)** Effects of VC and VD_3_ on body weight changes. **(B)** Body weight gain as a percentage of baseline weight for each group. **(C)** Pathologic examination of the liver by H&E staining, oil red O staining, and Masson staining. Representative images were captured. Scale bar, 100 μm. **(D)** The liver index, perirenal fat index, and epididymal fat index. **(E)** Serum levels of ALT and AST. **(F)** Serum lipids, including TC, TG, HDL-C, and LDL-C. **(G)** Fasting blood glucose. **(H)** Fasting serum insulin. **(I)** Insulin sensitivity index. **(J)** Homeostasis model assessment of insulin resistance. **(K)** Hepatic TC and TG levels. Data are shown as the means ± SDs (*n* = 12 mice/group), *p < 0.05, **p < 0.01, ***p < 0.001, ****p < 0.0001.

After 16 weeks of HFD feeding, all mice in the HFD group gained much more body weight than those in the ND group (Figures 1A, B). The liver specimens in the HFD group were larger and paler than those in the ND group (Figure 1C). However, the body weight and liver profile were significantly improved after treatment with VC and VD_3_ (Figures 1A–C). There was a significant increase in the liver fat, epididymal fat, and perirenal fat indexes in the HFD group (Figure 1D). VC and VD_3_ intervention attenuated the increases in the HFD-induced epididymal fat and perirenal fat indices (Figure 1D). However, the liver index was not affected by VC and VD_3_ intervention (Figure 1D).

As shown in Figure 1F, serum levels of TC, TG, and LDL-C in the HFD group were significantly higher than those in the ND group and were decreased by treatment with VC and VD_3_. However, the concentration of HDL-C was not significantly different among groups (*p* > 0.05). The HFD group displayed significantly higher FBG, insulin, and HOMA-IR and a lower ISI than those of the ND group (Figures 1G–J). VC intervention attenuated the HFD-induced increases in FBG and HOMA-IR, accompanied by opposite changes in ISI (Figures 1G–J). However, VD_3_ intervention had no effects on FBG, HOMA-IR or ISI (Figures 1G–J). VC intervention attenuated the HFD-induced increase in FBG and HOMA-IR, accompanied by opposite changes in ISI (Figures 1G–J). However, VD_3_ intervention had no effects on FBG, HOMA-IR or ISI (Figures 1G–J). Compared with the ND group, the HFD group mice exhibited markedly increased hepatic TC and TG levels, which were notably decreased after treatment with VC and VD_3_ (Figure 1K). Pathologic examination (H&E staining and ORO staining) further showed that MAFLD was successfully induced in the mice (Figure 1C). In the HFD group, lipid droplet vacuoles were abundant, cells were arranged irregularly, and the cytoplasm was loose. VC and VD_3_ treatment effectively improved these features (Figure 1C). Determination of serum transaminases revealed that there was no significant liver injury in HFD mice (Figure 1E). No positive results were found in Masson staining, indicating that the liver had not developed fibrosis at 16 weeks (Figure 1C). The above results clearly demonstrated that treatment with VC and VD_3_ could efficiently alleviate MAFLD in mice, and these effects in the VC group, VD group and VC + VD group were similar (all *p* > 0.05 among these three groups).

### VC and VD_3_ improved the gut microbiota in obese mice

**FIGURE 2:**
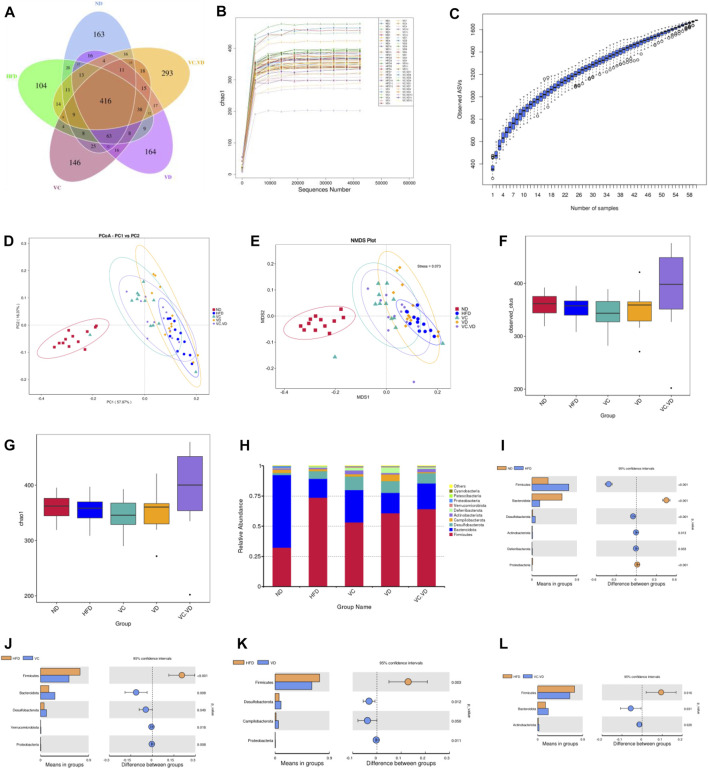
VC and VD_3_ improved the gut microbiota in obese mice. **(A)** Venn diagram of the ASVs from the gut microbiota. **(B)** Rarefaction curve of chao1. **(C)** Species accumulation boxplot. **(D–E)** Weighted UniFrac PCoA and NMDS analysis of the gut microbiota based on the ASV data. **(F–G)** Alpha diversity analyzed by the observed_otus and chao1 index. **(H)** Relative abundances at the phylum level. **(I–L)** Discrepancy in the gut microbiota composition between groups at the phylum level. Species with significant differences between the ND and HFD groups **(I)**, HFD and VC groups **(J)**, HFD and VD groups **(K)**, and HFD and VC + VD groups **(L)** were analyzed. A significant discrepancy was defined as *p* < 0.05 according to the *t*-test. (*n* = 12 mice/group).

To investigate the effects of VC and VD_3_ on the gut microbiota in obese mice, 16S rDNA amplicon sequencing in feces was performed. Dereplication was performed with DADA2 to procure a total of 1,695 ASVs from the 60 samples: 818 were in the ND group, 754 were in the HFD group, 811 were in the VC group, 831 were in the VD group, and 909 were in the VC + VD group (Figure 2A). There were 163 unique ASVs in the ND group, 104 unique ASVs in the HFD group, 146 unique ASVs in the VC group, 164 unique ASVs in the VD group, and 293 unique ASVs in the VC + VD group (Figure 2A). The rarefaction curve of chao1 and the species accumulation boxplot indicated that the sequencing depth and species richness were reasonable (Figures 2B, C). Based on the weighted UniFrac distance, principal coordinate analysis (PCoA) and non-metric multidimensional scaling (NMDS) plots showed distinguished clusters of microbiota composition for each group, revealing significant differences in the microbial community structure among the five groups (Figures 2D, E). The combination of VC and VD_3_ in mice improved the structure of the microbial community and increased its diversity and richness by α-diversity analysis [observed_otus and chao1 index] (Figures 2F, G). These data indicated that VC and VD_3_ played an essential role in regulating the gut microbiota profile.

As shown in Figure 2H, the main phyla in our research included Firmicutes, Bacteroidota, Desulfobacterota, Campilobacterota, Actinobacteriota, Deferribacterota, Verrucomicrobiota, Proteobacteria, Patescibacteria, and Cyanobacteria. The HFD significantly changed the composition of the gut microbiota by decreasing the relative abundances of Bacteroidota and Proteobacteria while increasing the relative abundances of Firmicutes, Desulfobacterota, Actinobacteriota, and Deferribacterota (Figure 2I). Under HFD conditions, VC supplementation reversed the abundances of Firmicutes, Bacteroidetes, and Proteobacteria (Figure 2J). Supplementation with VD_3_ reversed the abundances of Firmicutes and Proteobacteria (Figure 2K). VC + VD_3_ treatment reversed the abundances of Firmicutes and Bacteroidetes (Figure 2L).

**FIGURE 3:**
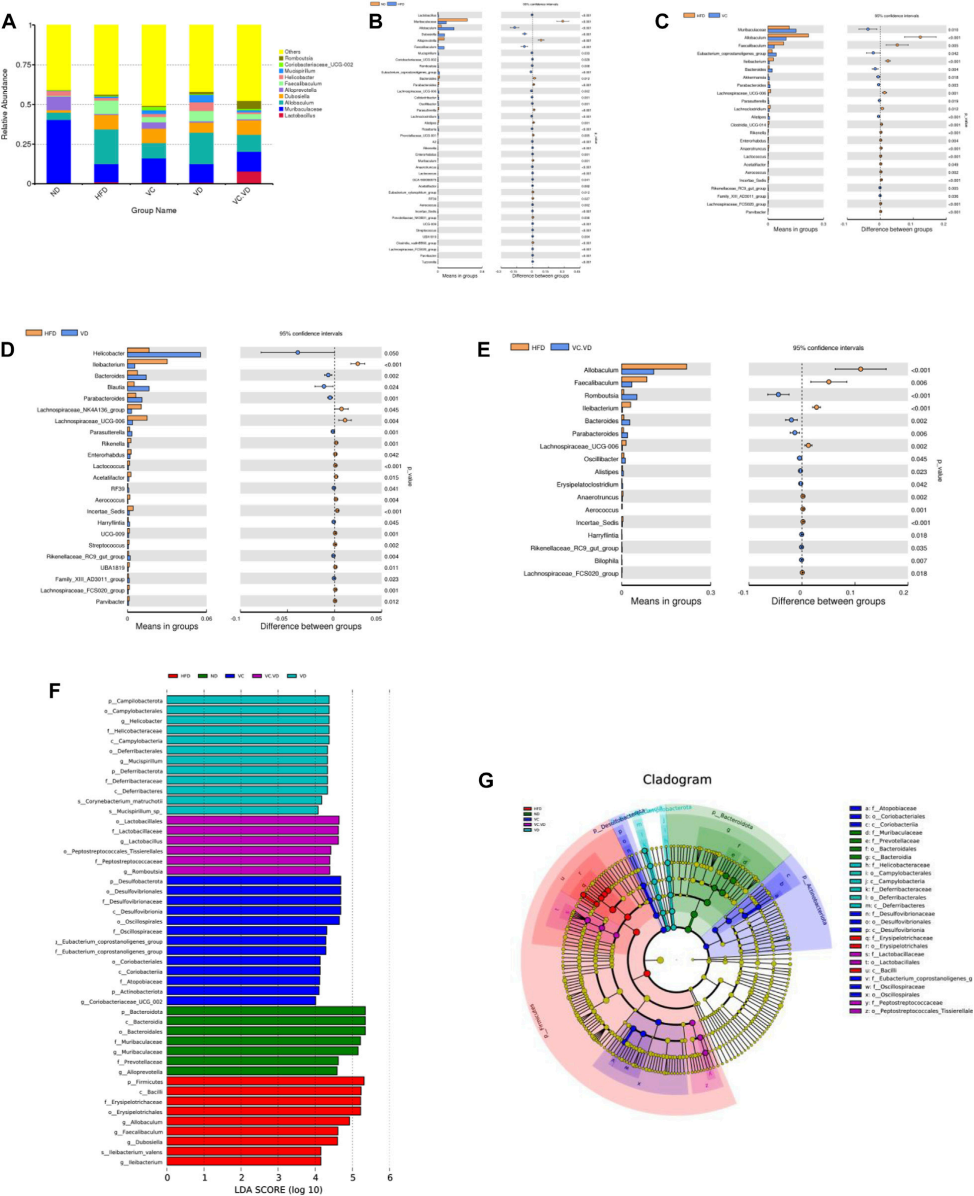
VC and VD_3_ improved the gut microbiota in obese mice. **(A)** Relative abundances at the genus level. **(B–E)** Discrepancy in the gut microbiota composition between groups at the genus level. Species with significant differences between the ND and HFD groups **(B)**, HFD and VC groups **(C)**, HFD and VD groups **(D)**, and HFD and VC + VD groups **(E)** were analyzed. A significant discrepancy was defined as *p* < 0.05 according to the *t*-test. **(F)** LEfSe analysis of the dominant biomarker taxa among the five groups. The threshold of the logarithmic score of LDA was 4.0. **(G)** Taxonomic cladogram obtained from LEfSe analysis by comparing the 5 groups. (*n* = 12 mice/group).

Next, the specific changes in the intestinal flora at the genus level were explored. The most abundant bacteria mainly included *Lactobacillus*, Muribaculaceae, Allobaculum, Dubosiella, Alloprevotella, Faecalibaculum, *Helicobacter*, Mucispirillum, Coriobacteriaceae_UCG-002, and Romboutsia. (Figure 3A). Detailed analysis at the genus level showed that HFD feeding significantly increased or decreased the relative abundances of many bacteria (Figure 3B). In particular, the HFD significantly increased the relative abundances of *Lactobacillus*, Allobaculum, Dubosiella, Faecalibaculum, Mucispirillum, Coriobacteriaceae_UCG-002, Romboutsia, and Lachnospiraceae_UCG-006, while it significantly decreased the relative abundances of Muribaculaceae, Alloprevotella, *Bacteroides*, Parabacteroides, and Parasutterella (Figure 3B). Under HFD conditions, VC intervention reversed the abundances of Allobaculum, Faecalibaculum, Muribaculaceae, Lachnospiraceae_UCG-006, *Bacteroides*, Parabacteroides, and Parasutterella (Figure 3C); VD_3_ supplementation reversed the abundances of Lachnospiraceae_UCG-006, *Bacteroides*, Parabacteroides, and Parasutterella (Figure 3D); and VC + VD_3_ intervention reversed the abundances of Allobaculum, Faecalibaculum, Lachnospiraceae_UCG-006, *Bacteroides*, and Parabacteroides (Figure 3E).

To identify the fecal microbial taxa that were most responsible for the differences in all groups, linear discriminate analysis effect size (LEfSe) analysis was conducted. When the LDA score was set at 4.0, we observed that the intestinal microbiota in each group was composed of specific bacterial taxa. Specifically, our results showed that there were 7, 9, 13, 12, and 6 significant differences in the ND, HFD, VC, VD, and VC + VD groups, respectively (Figure 3F). Analysis of cladograms also supported the marker taxa among the groups obtained from LEfSe analysis (Figure 3G). Overall, these results further demonstrated that VC and VD_3_ have a major role in shaping the intestinal flora in mice with MAFLD.

### VC and VD_3_ improved gut barrier integrity and endotoxemia in obese mice

**FIGURE 4:**
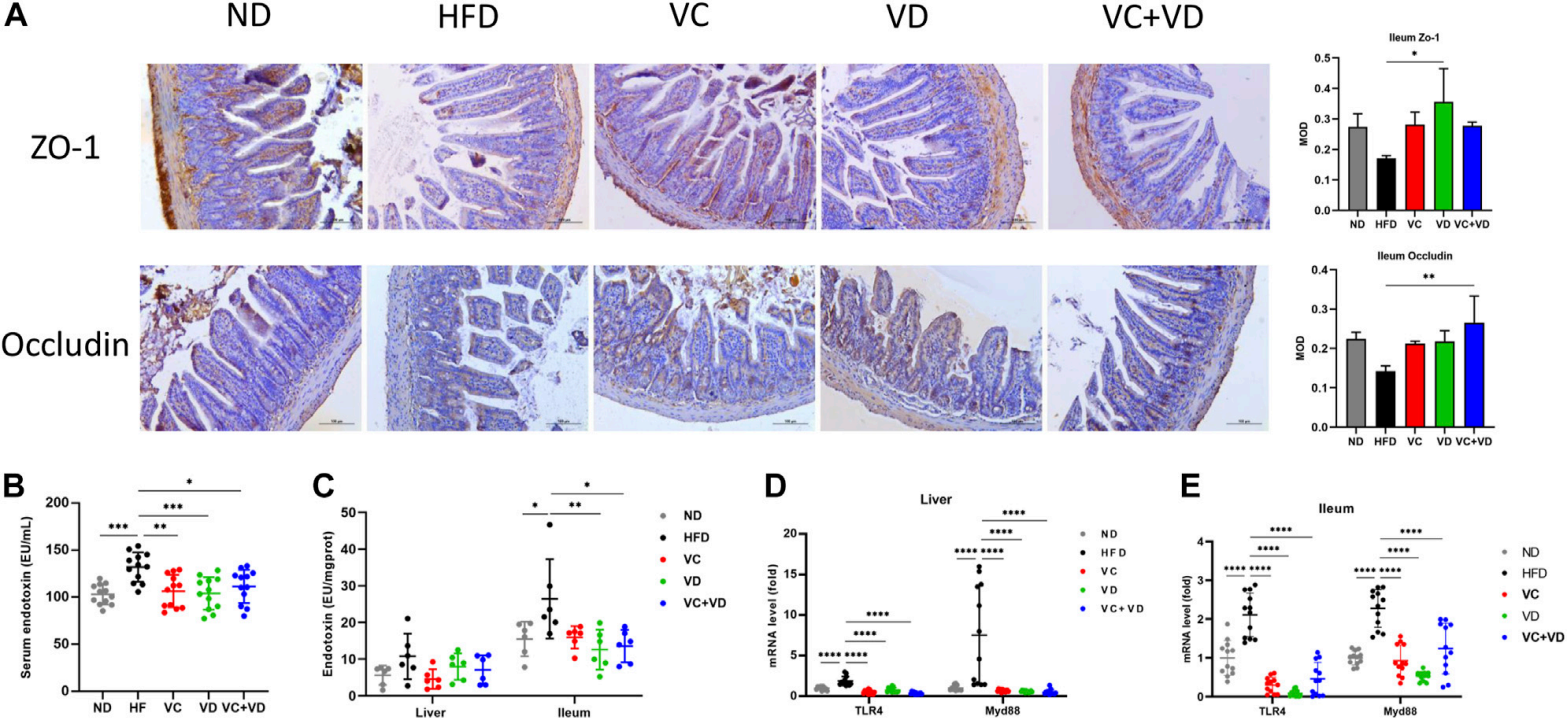
VC and VD_3_ improved gut barrier integrity and endotoxemia in obese mice. **(A)** Immunohistochemistry analysis of ZO-1 and Occludin in the ileum of mice. The brown dot indicates the target protein. Representative images were captured. Scale bar, 100 µm. **(B)** Serum levels of endotoxin. **(C)** Endotoxin levels in the liver and ileum. **(D)** Endotoxin-associated gene expression in the liver. **(E)** Endotoxin-associated gene expression in the ileum. The gene level in the ND group was set as 1, and the relative fold increases were determined by comparison with the ND group. Data are shown as the means ± SDs (*n* = 12 mice/group), *p < 0.05, **p < 0.01, ***p < 0.001, ****p < 0.0001.

It has been fully verified that a disordered intestinal microbiota can destroy the integrity of enterocyte tight junctions, leading to endotoxin flowing into the blood and causing systemic inflammation (Zhao et al., 2013; Luck et al., 2015). We thus determined the protein expression of the tight junction proteins ZO-1 and Occludin in the ileum using immunohistochemistry. As shown in Figure 4A, HFD feeding dramatically reduced the expression of ZO-1 and Occludin, and this reduction was reversed by VC or VD_3_ to a certain degree (Figure 4A). Furthermore, the endotoxin levels in the serum and ileum in the HFD group were significantly increased compared with those in the ND group but were significantly decreased after VC and VD_3_ intervention (Figures 4B, C). Although there were no significant differences among all the groups, the liver endotoxin level was increased in the HFD group compared with the other groups (Figure 4C). Endotoxin-associated TLR4 and Myd88 mRNA expression in the ileum and liver in the HFD group was significantly increased compared with that in the ND group (Figures 4D, E). In contrast, the levels of these endotoxin-associated gene mRNAs were all significantly reduced by VC and VD_3_ intervention compared with levels in the HFD group (Figures 4D, E). Taken together, these results indicate that VC and VD_3_ could improve intestinal barrier function in MAFLD mice.

### VC and VD_3_ ameliorated oxidative stress and inflammation in the gut-liver axis in obese mice

**FIGURE 5:**
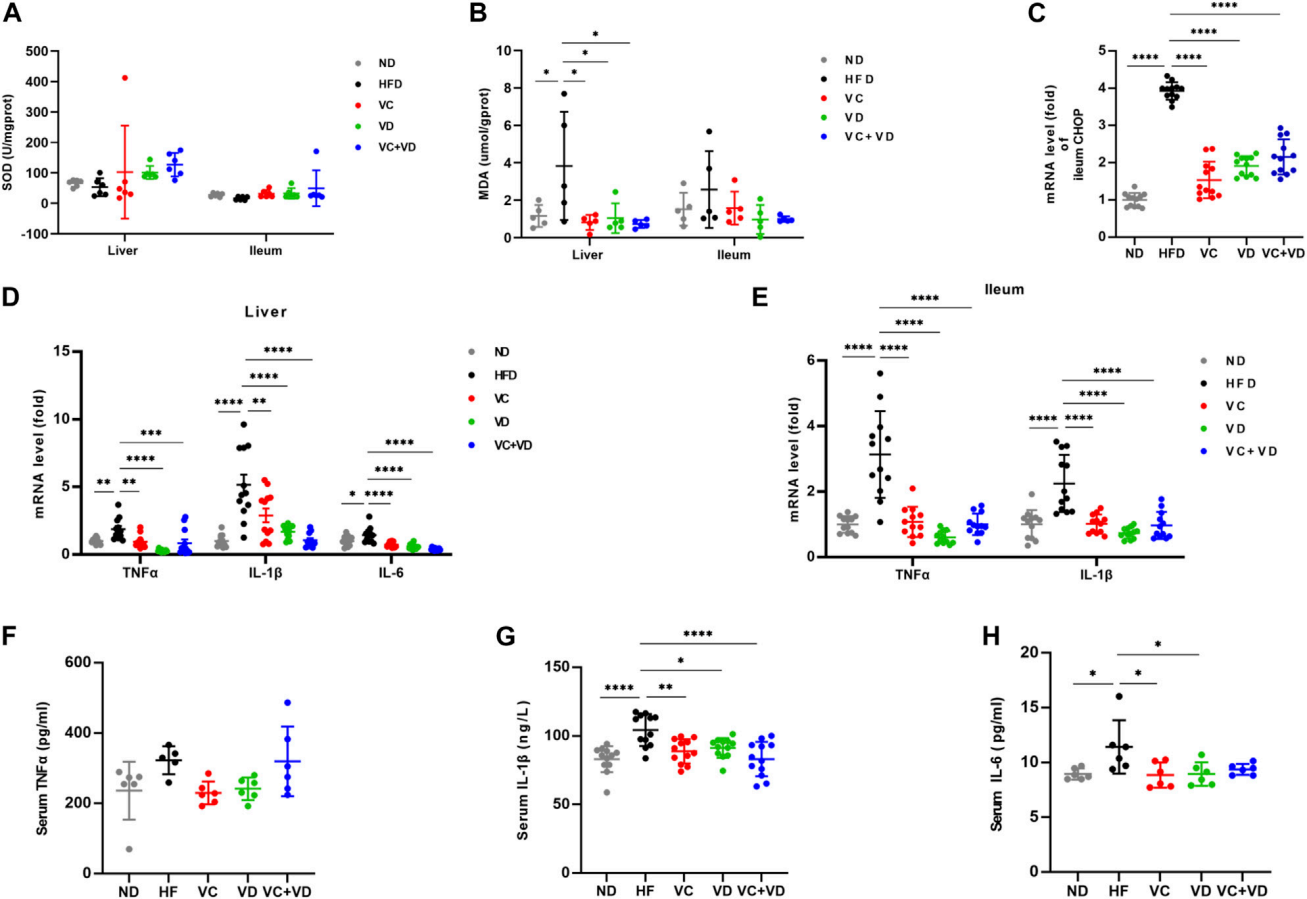
VC and VD_3_ ameliorated oxidative stress and inflammation in the gut-liver axis in obese mice. **(A)** SOD of the liver and ileum. **(B)** MDA of the liver and ileum. **(C)** Gene expression of C/EBP-homologous protein in the ileum of mice. **(D)** Proinflammation-associated gene expression in the liver. **(E)** Proinflammation-associated gene expression of the ileum. The gene level in the ND group was set as 1, and the relative fold increases were determined by comparison with the ND group. **(F)** Serum TNFα. **(G)** Serum IL-1β. **(H)** Serum IL-6. Data are shown as the means ± SDs (*n* = 12 mice/group), *p < 0.05, **p < 0.01, ***p < 0.001, ****p < 0.0001.

It is well known that obesity and hepatic steatosis are correlated with liver oxidative stress. We evaluated the effects of VC and VD_3_ on oxidative stress in the liver and ileum by examining the levels of SOD and MDA (Figures 5A, B). The liver MDA content in the HFD group was significantly higher than that in the ND group (Figure 5B). As expected, compared with HFD-fed mice, VC and VD_3_ significantly reduced the content of MDA (Figure 5B). Although there were no significant differences among all the groups, the SOD levels (Figure 5A) in the liver and ileum in the HFD mice were lower than those in the other groups, and the ileum MDA content (Figure 5B) was higher than that in the other groups. In addition, the mRNA expression of C/EBP-homologous protein (CHOP) was dramatically increased in the HFD group compared with the other groups, indicating that oxidative stress-induced cell apoptosis in the ileum was attenuated by VC and VD_3_ intervention (Figure 5C). Liver oxidative stress is related to a low degree of chronic inflammation. We investigated the effects of VC and VD_3_ on proinflammatory cytokines, such as TNF-α, IL-1β, and IL-6. The mRNA expression of proinflammatory cytokines was widely enhanced in the ileum and liver in the HFD group, which was markedly suppressed by VC and VD_3_ intervention (Figures 5D, E). Serum inflammatory cytokine levels as assessed by ELISA were also unexpectedly enhanced in the HFD group (Figures 5F–H). In contrast, these inflammatory cytokine levels were reduced to a certain degree by VC and VD_3_ supplementation (Figures 5F–H). These results indicated that VC and VD_3_ significantly reduced systemic low-grade inflammation.

### Effect of VC and VD_3_ on liver metabolomic profiling in obese mice

**FIGURE 6:**
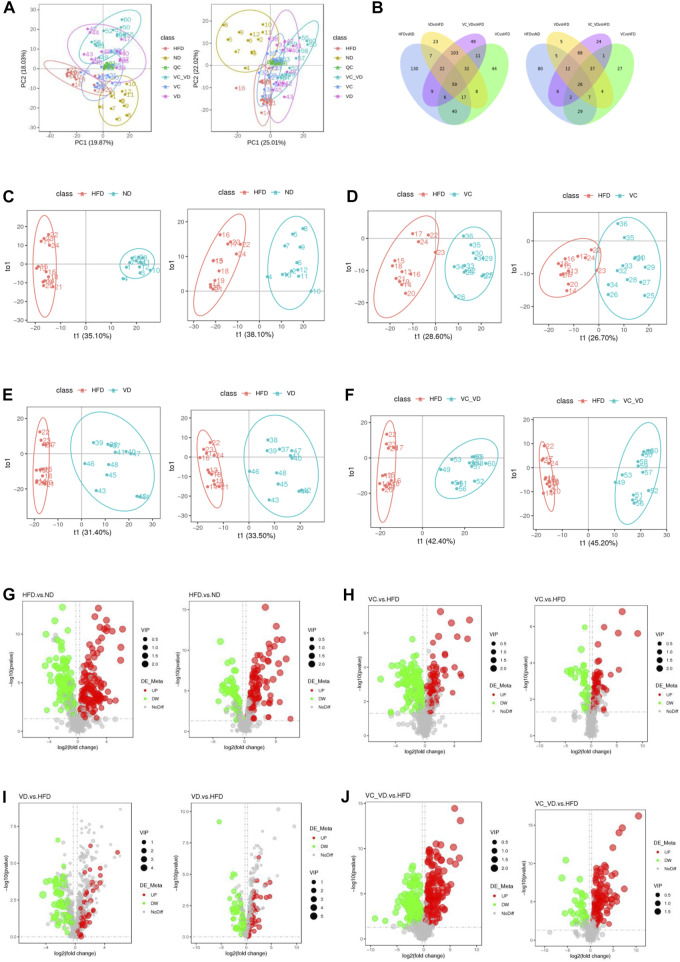
Effect of VC and VD_3_ on liver metabolomic profiling in obese mice. **(A)** PCA score plots of the different groups in positive polarity (left) and negative polarity (right) modes. The quality control (QC) sample was prepared by mixing equal volumes of all the samples. **(B)** Venn diagrams displaying the number of differentially abundant metabolites that overlapped in the HFD vs. ND, VC vs. HFD, VD vs. HFD, and VC + VD vs. HFD comparisons in positive polarity (left) and negative polarity (right) modes. **(C)** OPLS-DA score plots of the HFD and ND groups in positive polarity (left) and negative polarity (right) modes. **(D)** OPLS-DA score plots of the HFD and VC groups in positive polarity (left) and negative polarity (right) modes. **(E)** OPLS-DA score plots of the HFD and VD groups in positive polarity (left) and negative polarity (right) modes. **(F)** OPLS-DA score plots of the HFD and VC + VD groups in positive polarity (left) and negative polarity (right) modes. **(G)** Volcano plots showing the dysregulated features between the HFD and ND groups in positive polarity (left) and negative polarity (right) modes. **(H)** Volcano plots for the VC and HFD groups in positive polarity (left) and negative polarity (right) modes. **(I)** Volcano plots for the VD and HFD groups in positive polarity (left) and negative polarity (right) modes. **(J)** Volcano plots for the VC + VD and HFD groups in positive polarity (left) and negative polarity (right) modes.

To further explore the possible mode of action of VC and VD_3_ on the liver physiological and metabolic response in MAFLD mice, liver untargeted metabolomics was performed by LC‒MS/MS, and a total of 1,201 metabolites were detected (the number of positive and negative polarity modes was 771 and 430, respectively, Table 2) in the metabolomics data. As shown in Figure 6A, a tight clustering of principal component scores in QC samples indicates a stable and reliable instrument and results. The samples from different groups were divided into significant clusters in the principal component analysis (PCA) score plots of positive and negative modes, showing that differentiation among the five groups was observed in liver metabolite profiles (Figure 6A). Venn diagrams displayed the overlap of the differential metabolites in HFD vs. ND, VC vs. HFD, VD vs. HFD, and VC + VD vs. HFD (Figure 6B). Orthogonal partial least squares discrimination analysis (OPLS-DA) was used to examine the differences between the HFD and the other groups. In the score plots, metabolic differentiation was evident between them (Figures 6C–F).

**TABLE 2:** Differential metabolite screening results.

Compared groups	Num. of total ident	Num. of total sig	Num. of Sig.Up	Num.of Sig.down
HFD.vs. ND_pos	771	290	167	123
VC.vs. HFD_pos	771	217	101	116
VD.vs. HFD_pos	771	271	161	110
VC_VD.vs. HFD_pos	771	291	182	109
HFD.vs. ND_neg	430	167	106	61
VC.vs. HFD_ neg	430	133	92	41
VD.vs. HFD_ neg	430	164	125	39
VC_VD.vs. HFD_neg	430	176	140	36

The threshold is set to VIP >1.0, FC > 1.2 or FC < 0.833, and p-value < 0.05.

pos, positive polarity mode; neg, negative polarity mode; Num of Total Ident, total number of identification results of metabolites; Num of Total Sig, total number of metabolites with significant difference; Num of Sig Up, total number of metabolites significantly upregulated; Num of Sig down, total number of metabolites significantly downregulated.

The screening results of differential metabolites (the threshold was set to VIP >1.0, FC > 1.2 or FC < 0.833, and *p*-value < 0.05) are shown in Table 2. Compared with ND mice, a different metabolism profile was displayed in HFD mice, as indicated by the detection of 290 metabolites with significant differences in positive polarity mode (167 upregulated and 123 downregulated) (Figure 6G, left panel) and 167 metabolites with significant differences in negative polarity mode (106 upregulated and 61 downregulated) (Figure 6G, right panel). Compared with HFD mice, a different metabolism profile was observed in mice treated with VC, as indicated by the detection of 217 metabolites with significant differences in positive polarity mode (101 upregulated and 116 downregulated) (Figure 6H, left panel) and 133 metabolites with significant differences in negative polarity mode (92 upregulated and 41 downregulated) (Figure 6H, right panel). Compared with HFD mice, a different metabolism profile was observed in mice treated with VD_3_, as indicated by the detection of 271 metabolites with significant differences in positive polarity mode (161 upregulated and 110 downregulated) (Figure 6I, left panel) and 164 metabolites with significant differences in negative polarity mode (125 upregulated and 39 downregulated) (Figure 6I, right panel). Compared with HFD mice, mice treated with VC + VD_3_ showed a different metabolism profile, as indicated by the detection of 291 metabolites with significant differences in positive polarity mode (182 upregulated and 109 downregulated) (Figure 6J, left panel) and 176 metabolites with significant differences in negative polarity mode (140 upregulated and 36 downregulated) (Figure 6J, right panel). These metabolites could be classified into enzenoids, lipids and lipid-like molecules, nucleosides, nucleotides and analogs, organic acids and derivatives, organic nitrogen compounds, organic oxygen compounds, organoheterocyclic compounds, and phenylpropanoids and polyketides.

**FIGURE 7:**
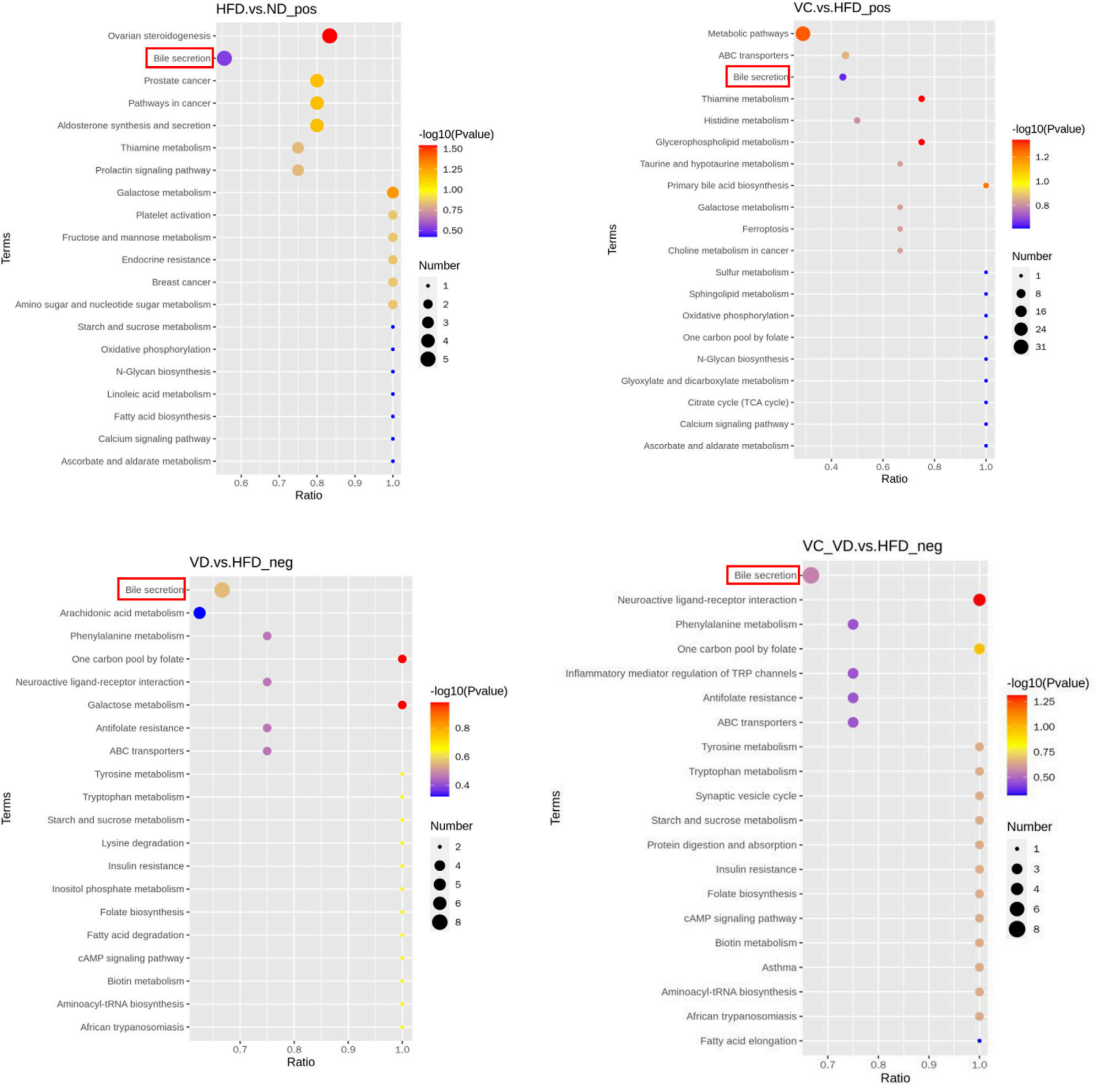
KEGG_Enrich.scatterplot. KEGG_Enrich.scatterplot (top 20) of the HFD vs. ND, VC vs. HFD, VD vs. HFD, and VC + VD vs. HFD groups; pos, positive polarity mode; neg, negative polarity mode. (*n* = 12 mice/group).

To gain a deeper understanding of the differential metabolite changes upon VC and VD_3_ supplementation, metabolic pathway enrichment was performed using the Kyoto Encyclopedia of Genes and Genomes (KEGG) database. The top 20 annotated KEGG pathways showed that the bile secretion pathway might be closely associated with the effects of VC and VD_3_ on MAFLD (Figure 7). Specifically, the changes in the liver metabolism profile are as follows: compared with ND mice, HFD mice exhibited downregulated choline, taurocholic acid (TCA), and lithocholic acid (LCA) and upregulated hydrocortisone and bilirubin; compared with HFD mice, mice treated with VC exhibited downregulated bilirubin and upregulated choline, TCA, and glutathione; compared with HFD mice, mice treated with VD_3_ had downregulated uric acid and upregulated chenodeoxycholic acid (CDCA), prostaglandin E2, serotonin, deoxycholic acid (DCA), cholic acid (CA), folic acid, and thromboxane B2; compared with HFD mice, the mice treated with VC + VD_3_ also had downregulated uric acid and upregulated CDCA, prostaglandin E2, serotonin, DCA, CA, folic acid, and thromboxane B2 (Table 3).

**TABLE 3:** The changes of liver metabolism profile as for bile secretion pathways.

Liver metabolite	HFD vs. ND	VC vs. HFD	VD vs. HFD	VC + VD vs. HFD	Polarity mode
Choline	↓	↑			positive
Taurocholic acid	↓	↑			positive
Lithocholic acid	↓				positive
Hydrocortisone	↑				positive
Bilirubin	↑	↓			positive
Glutathione		↑			positive
Uric acid			↓	↓	negative
Chenodeoxycholic Acid			↑	↑	negative
Prostaglandin E2			↑	↑	negative
Serotonin			↑	↑	negative
Deoxycholic acid			↑	↑	negative
Cholic acid			↑	↑	negative
Folic acid			↑	↑	negative
Thromboxane B2			↑	↑	negative

ND, normal diet group; HFD, high-fat diet group; VC, Vitamin C group; VD, Vitamin D3 group; VC + VD, Vitamin C + Vitamin D3 group.

### VC and VD_3_ improved bile acid metabolism in the gut-liver axis in obese mice

**FIGURE 8:**
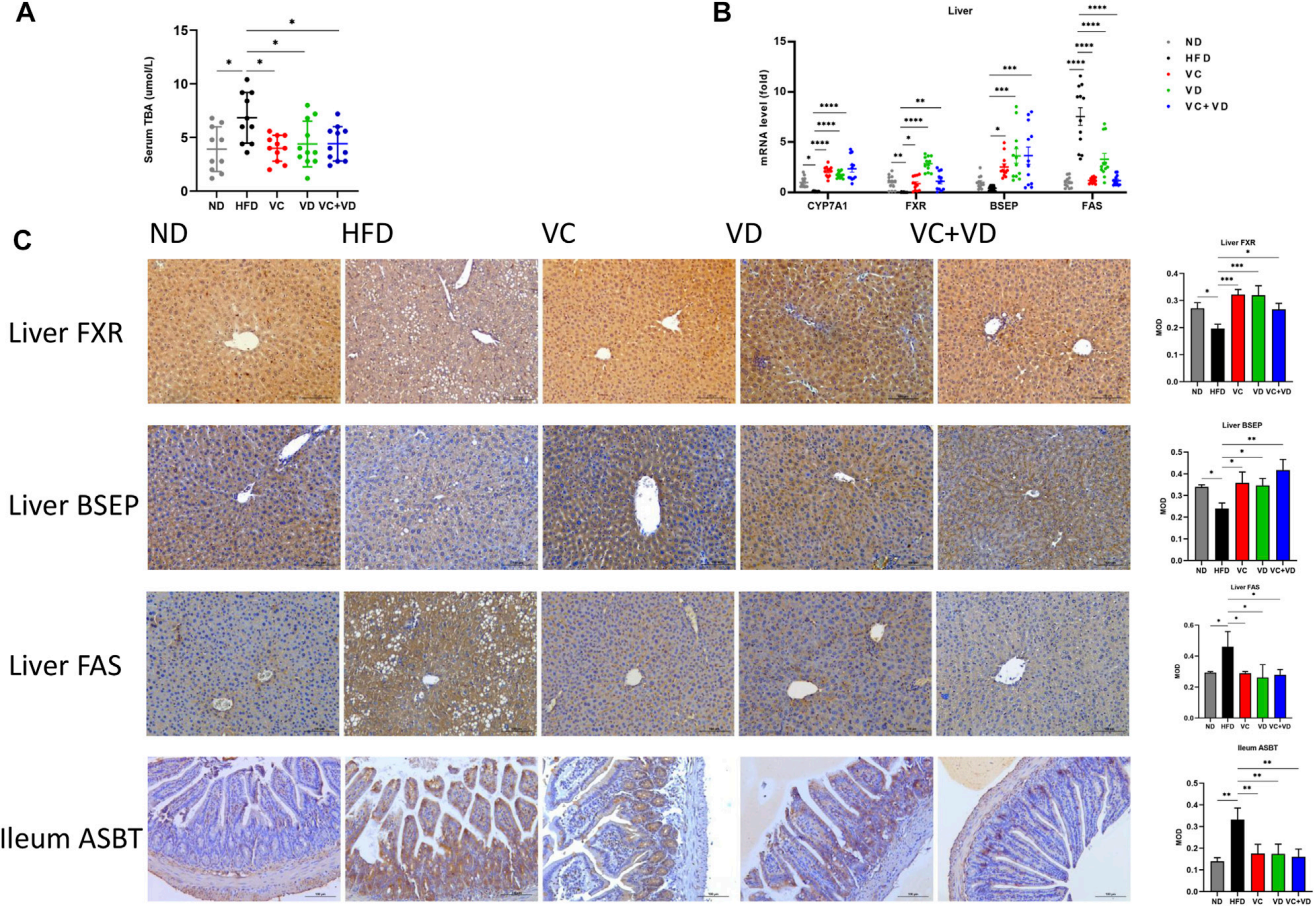
VC and VD_3_ attenuated bile acid metabolism dysfunction in the gut-liver axis in obese mice. **(A)** Serum levels of total bile acid. **(B)** Expression of mRNAs involved in the regulation of the synthesis and transport of bile acids and fatty acid synthase (FAS) in the liver. **(C)** Immunohistochemistry analysis of FXR, BSEP, and FAS in the liver and ASBT in the ileum of mice. The brown dot indicates the target protein. Representative images were captured. Scale bar, 100 µm. Data are shown as the means ± SDs (*n* = 12 mice/group), *p < 0.05, **p < 0.01, ***p < 0.001, ****p < 0.0001.

We then explored the regulatory effect of VC and VD_3_ on bile secretion in the gut-liver axis. Serum TBA levels in the HFD group were significantly higher than those in the ND group and were decreased by treatment with VC and VD_3_ (Figure 8A). As shown in Figure 8B, gavage of VC and VD_3_ in HFD mice increased the expression of the bile acid synthesis-related gene CYP7A1 and bile acid transportation-related genes in the liver, such as FXR and BSEP. The immunohistochemical results were in line with the tendencies of the mRNA expression changes (Figure 8C). Furthermore, gavage of VC and VD_3_ in HFD mice decreased the expression of bile acid reflux regulatory ASBT in the gut (Figure 8C). Ultimately, VC and VD_3_ treatment decreased the mRNA and protein expression of FAS in the liver (Figures 8B, C). These results suggested that VC and VD_3_ are beneficial for bile acid metabolism in the gut-liver axis.

## Discussion

Our study investigated the prophylactic effects of VC and VD_3_ in MAFLD and its underlying molecular mechanism in a HFD-induced obese mouse model. The present study indicated that MAFLD mice treated with VC and VD_3_ exhibited improved obesity, dyslipidemia, insulin resistance (IR), hepatic steatosis, and inflammation. However, VC and VD_3_ showed high similarity with respect to their anti-MAFLD actions, and there was no significant difference between the single drug groups and their combination. Notably, VC and VD_3_ treatment reshaped the composition of the gut microbiota, improved the integrity of the gut barrier, and ameliorated oxidative stress and inflammation of the gut-liver axis. Furthermore, VC and VD_3_ treatment in obese mice reduced bile acid salt reflux into the liver from the gut, further activating bile acid synthesis-related CYP7A1, the bile acid receptor FXR, and bile acid transportation-related BSEP in the gut-liver axis and improving bile secretion, thus decreasing FAS expression in the liver and efficiently ameliorating MAFLD in mice. Our study provided new clues to explain the prophylactic effect of VC and VD_3_ on MAFLD. To our knowledge, this is the first study to explore the potential mechanism of VC in combating MAFLD and to compare its beneficial role with that of VD_3_. However, a note of caution should be acknowledged with regard to translating results obtained in animal models of MAFLD into the clinic, because these models do not completely mirror human disease (Flessa et al., 2022).

Recently, it has been shown that VC intake in MAFLD patients is lower than the recommended dietary allowance in some epidemiological studies, indicating that there is a correlation between VC deficiency and MAFLD (Ipsen et al., 2014). An appropriate increase in VC intake has been shown to be associated with a significantly lower risk of developing IR and MAFLD (Donin et al., 2016; Wei et al., 2016). The effect of VC in HFD-fed mice may be to increase the expression of adiponectin and adiponectin receptor II, which is related to various metabolic disorders such as obesity and diabetes (Yamauchi et al., 2014; Zeng et al., 2020). Not surprisingly, MAFLD mice treated with VC had reduced body weight and white adipose tissue index and improved dyslipidemia and IR in our study. As an antioxidant, VC has been shown to inhibit oxidative stress and inflammation, improve IR, regulate lipid metabolism, and protect the liver (Padayatty et al., 2003; Ellulu et al., 2015), which was consistent with our findings. Prophylactic supplementation of VC to HFD-fed mice may slow the development of MAFLD.

VD plays a prominent role not only in calcium/phosphorus regulation and bone homeostasis but also in immunity, inflammation, proliferation, and differentiation (Nagpal et al., 2005; Baeke et al., 2010). Growing clinical and basic research evidence shows that low VD levels are closely related to the characteristics of metabolic syndrome, including MAFLD and diabetes (Erbas et al., 2014; Barrea et al., 2019; Barchetta et al., 2020). VD deficiency commonly occurs in 55% of MAFLD patients (Barchetta et al., 2011; Wang et al., 2015; Kim et al., 2017). Therefore, an increasing number of scholars have begun to study the effect of VD on MAFLD. As a result of preventive VD treatment, lipogenic and inflammatory genes were reduced in the liver, and intestinal inflammation was reduced as well (Jahn et al., 2019). Makishima et al. showed that the vitamin D receptor also functions as a receptor for the secondary bile acid lithocholic acid, which is hepatotoxic and a potential enteric carcinogen (Makishima et al., 2002). However, Dong et al. reported that activation of the VD receptor by VD ligands in liver macrophages ameliorated liver inflammation, steatosis, and IR in mice (Dong et al., 2020). In line with previous studies, we observed that VD supplementation exerts a positive effect on obesity, oxidative stress, inflammation, and hyperlipidemia in MAFLD mice. However, VD supplementation did not markedly improve IR and hyperglycemia in the current study. Schmidt et al. showed that dietary VD inhibits bile acid synthesis by repressing hepatic expression of CYP7A1 (Schmidt et al., 2010). The results of our experiment seem to be inconsistent with those of some previous studies. It remains to be determined whether these different results are due to the delivery method, the timing of administration or the different models of MAFLD.

Previous studies have indicated that an increase in the Firmicutes to *Bacteroides* ratio (F/B) is closely associated with obesity (Fontane et al., 2018; Rao et al., 2021). In our study, it was found that a HFD significantly altered the intestinal flora composition by increasing the abundance of Firmicutes and decreasing that of Bacteroidetes, resulting in an increase in the F/B ratio. Treatment with VC or VD_3_ showed an opposite tendency to reduce the F/B ratio. The major significant findings in our experiment included a decrease in the abundances of Bacteroidota and Proteobacteria and an increase in the abundances of Firmicutes, Desulfobacterota, Actinobacteriota, and Deferribacterota at the phylum level in HFD-induced obese mice. Consistent with the study by Zheng et al., we also found that Proteobacteria decreased markedly in the HFD-fed group (Zheng et al., 2017). However, the VC and VD_3_ treatments significantly increased the abundance of Proteobacteria. Other studies have shown that an increase in the abundances of Actinobacteriota and Deferribacterota is involved in the progression of MAFLD, in accordance with our results (Li et al., 2020b; Wu et al., 2020). In addition, VC also increased the abundance of Verrucomicrobiota, and VD reduced the abundance of Patescibacteria. This change trend is beneficial to MAFLD (Leung et al., 2016; Wu et al., 2020). The HFD significantly increased the relative abundances of *Lactobacillus*, Allobaculum, Dubosiella, Faecalibaculum, Mucispirillum, Coriobacteriaceae_UCG-002, Romboutsia, and Lachnospiraceae_UCG-006, while it significantly decreased the abundances of Muribaculaceae, Alloprevotella, *Bacteroides*, Parabacteroides, and Parasutterella at the genus level compared to the ND group, which was partially consistent with previous studies (Zeng et al., 2019; Li et al., 2020b; Rao et al., 2021). Interestingly, VC intervention reversed the abundances of Allobaculum, Faecalibaculum, Muribaculaceae, Lachnospiraceae_UCG-006, *Bacteroides*, Parabacteroides, and Parasutterella; VD_3_ intervention reversed the abundances of Lachnospiraceae_UCG-006, *Bacteroides*, Parabacteroides, and Parasutterella; and VC + VD_3_ intervention reversed the abundances of Allobaculum, Faecalibaculum, Lachnospiraceae_UCG-006, *Bacteroides*, and Parabacteroides.

Bile acid is synthesized primarily in the liver, is transported to the gallbladder and intestine, and then is refluxed back into the liver (Wahlstrom et al., 2016). On the one hand, probiotics play an important role in regulating nuclear receptor activation and maintaining the normal bile acid pool; on the other hand, the steady state of the bile acid pool is important for maintaining the balance of the intestinal flora (Chen et al., 2019). The gut-liver axis is therefore characterized by bidirectional interaction between the gut microbiota and bile acids. Disorder of the bile acid pool is related to multiple diseases, including obesity and MAFLD (Devkota et al., 2012; Jiao et al., 2018). Due to the rapid progress in the fatty liver–bile acid field, a large sample of liver biopsy-proven MAFLD patient samples were tested by bile acid spectroscopy, and the secondary bile acid levels were found to be significantly increased in MAFLD patients (Liu et al., 2023). Similarly, we found that the bile secretion pathway might be closely associated with the effects of VC and VD_3_ on MAFLD by liver metabolomics analysis. Compared to ND mice, HFD mice had decreased TCA and LCA levels, which could activate the bile acid receptors FXR and TGR5 (Wahlstrom et al., 2016). After treatment with VC and VD_3_, the levels of some bile acids were increased, indicating that VC and VD_3_ increased CYP7A1 expression levels. As a rate-limiting enzyme in bile acid synthesis, CYP7A1 can promote cholesterol conversion into bile acids, thus lowering blood cholesterol levels (Wahlstrom et al., 2016). The increase in bile acid levels activated FXR and TGR5 and then modulated lipogenesis and lipid oxidation in MAFLD (Han et al., 2019; Clifford et al., 2021). Previous studies have shown that FXR can directly regulate FAS transcription (Matsukuma et al., 2006; Shen et al., 2011). Further experimental verification suggested that gavage of VC and VD_3_ in HFD mice decreased the expression levels of ASBT in the gut and increased the expression levels of CYP7A1, FXR, and BSEP in the liver, resulting in an improvement in bile acid metabolism in the gut-liver axis. Ultimately, VC and VD_3_ treatment decreased FAS mRNA and protein expression in the liver.

The fact that all 3 therapies (VC, VD_3_, and VC + VD_3_) had mostly similar effects on MAFLD parameters implies a common downstream pathway that is not specific to either VC or VD_3_ or their receptors. Nevertheless, our results allow us to draw the following conclusion: either VC or VD_3_ can regulate the intestinal flora and bile acid metabolism and then further regulate lipid metabolism through specific biochemical and metabolic pathways. Perhaps this is related to bile acid/FXR and its downstream pathway. However, the changes in specific bacterial species and the bile acid composition are still different in the different groups. Therefore, we will focus on the effects of specific bacterial species and bile acid components on MAFLD in the future.

The current study has several limitations. (i) As of August 2022, it was not clear if a consensus to rename NAFLD to MAFLD had been reached. However, because this term has been widely recognized, it is still used in the present manuscript. (ii) The mechanism through which VC and VD_3_ function in the treatment of MAFLD in a cell model that mimics liver steatosis has not been revealed, which makes it difficult to study the gut-liver axis *in vitro*. (iii) We did not quantify the liver bile acid composition (proportion of primary to secondary bile acids), which would provide more insight into the circulation and reabsorption of bile acids. Thus, we will conduct metabolomics analysis for the quantification of bile acids in future mechanistic research. (iv) Finally, we did not examine the levels of gene expression and enzyme activity of bile acid converting enzymes in the gut microbiomes, which is a future direction for understanding the production of secondary bile acids in MAFLD. Nevertheless, based on the evidence presented here, we reasonably believe that VC and VD_3_ ameliorate MAFLD in mice, the mechanism of which is closely related to their modulating effect on the gut microbiota and bile acids in the gut-liver axis.

## Conclusion

In summary, the study demonstrated that both VC and VD_3_ could modulate intestinal microbes, improve the intestinal barrier, ameliorate oxidative stress and inflammation in the gut-liver axis, attenuate bile acid metabolism dysfunction in the gut-liver axis, and alleviate MAFLD in the HFD-induced obese mouse model. Since VC, VD_3_ and VC + VD_3_ administration showed great similarity in improving MAFLD through the mechanism of action mentioned above, it is recommended that only one of these vitamins be used alone. These findings suggest that VC and VD_3_ may prove to be effective treatments for MAFLD by regulating the gut microbiota and bile acid metabolism *via* the gut-liver axis, which may be the drug target of future MAFLD intervention. However, more evidence is needed to verify the mechanism through which VC and VD_3_ modulate the gut microbiota and liver bile acid metabolism in the context of MAFLD.

## Data Availability

The datasets presented in this study can be found in online repositories. The names of the repository/repositories and accession number(s) can be found below: NCBI’s BioProject database; accession PRJNA903082.
